# THE BENEFIT OF COOPERATIVE EXTENSION FOR OLDER ADULT MENTAL HEALTH AND FUTURE GERONTOLOGICAL RESEARCH OPPORTUNITIES

**DOI:** 10.1093/geroni/igae098.1697

**Published:** 2024-12-31

**Authors:** Naomi Meinertz, Kate Perepezko, David Buys

**Affiliations:** Chair; Co-Chair; Discussant

## Abstract

Mental health concerns are a gerontological research priority; however, older adults who would benefit most from research in this area (e.g., rural, socially isolated) are often underrepresented in research. With representation in every U.S. state, the Cooperative Extension (Extension) system is ideally situated to work with researchers to reach and positively impact older adults. However, despite a strong history of success in bridging research and practice, Extension is an often-overlooked asset. The main objective of this symposium is to highlight exemplary partnerships between academic faculty and Extension systems to positively impact older adult mental health. The first presentation will discuss the results of a systematic review highlighting studies that employed Community-based Participatory Research methods with the Extension system to positively impact older adult mental health. The second presentation will share the process of culturally adapting the National Council on Aging’s ‘Aging Mastery Program’ through the Extension system and the impact on older participants’ feelings of social connectedness. The third presentation will present the findings of a novel program, ‘Rightsizing’, that reduces hoarding behaviors of older adults by addressing their emotional connection to their possessions. The final presentation will identify the process of partnering with a Cooperative Extension system that implements a multi-state physical activity program, ‘Stay Strong Stay Healthy’, to improve older adult physical and psychosocial outcomes. This symposium will conclude with a discussion led by Dr. David Buys connecting each of the presentations and emphasizing the role of the national Extension system in improving older adult mental health outcomes. Mental Health Practice and Aging Interest Group Sponsored Symposium

